# Beyond the knee: Why hip examination matters in patients with knee pain

**DOI:** 10.1002/jeo2.70525

**Published:** 2025-11-14

**Authors:** Bert Cornelis, Gilles Van Eetvelde, Sigurd Uyttebroek, Maxence Vandekerckhove, Jan Vanlommel, Pieter‐Jan Vandekerckhove

**Affiliations:** ^1^ Department of Orthopedics and Traumatology University Hospital Ghent Gent Belgium; ^2^ Department of Orthopedics and Traumatology AZ Sint Lucas Hospital Gent Belgium; ^3^ Department of Orthopedics and Traumatology Orthoclinic, AZ Sint‐Jan Hospital Bruges Belgium

**Keywords:** hip pain, incidence, knee examination, knee pain, referred knee pain

## Abstract

**Purpose:**

Knee pain is a common reason for consulting an orthopaedic surgeon. It has a wide variety of aetiology, of which one is hip pathology. This phenomenon is called referred knee pain. While it is well‐known, it has not been thoroughly described from the perspective of the knee surgeon. With this observational study, the importance of routinely including clinical examination of the hip during knee examination is highlighted.

**Methods:**

A retrospective analysis was performed of all patients with knee pain who consulted the orthopaedic service for the first time during a period of 1 year. Both self‐referred patients and those referred by other physicians were included. A full anamnesis and physical examination of the knee and hip were conducted. If hip pathology was suspected, a referral was made to a hip surgeon. Treatment was provided accordingly. A subjective improvement in pain or, when available, a ≥50% reduction in visual analogue scale score was considered a positive diagnosis of ‘referred knee pain’.

**Results:**

Of the 1000 patients presenting for first‐time consultation, 185 (18.5%) were referred to hip specialists for further assessment. Among the referred patients, 157 (84.9%) were found to have hip pathology, while 27 (14.6%) exhibited a combined hip and knee pathology. The most prevalent diagnoses included femoroacetabular impingement (22.9%) and osteoarthritis (58%). The predominant treatment modalities consisted of total hip arthroplasty (7.8%), physiotherapy (29.9%), and corticosteroid injections (54.1%). Referred knee pain was diagnosed in 137 (13.7%) of the cohort, with a higher prevalence in females (15.2% vs. 11.8% in males; *p* = 0.03).

**Conclusion:**

With 13.7% of knee pain patients presenting with an underlying hip pathology, referred knee pain is a common and often overlooked phenomenon in the daily practice of a full‐time knee surgeon. A complete history and physical examination including the hip is obligatory for every knee exam.

**Level of Evidence:**

Level III, retrospective observational cohort study.

**Abbreviations**
FAI: femoroacetabular impingement
PAO: peri‐acetabular impingement
THA: total hip arthroplasty

## INTRODUCTION

Knee pain is a frequent complaint leading patients to seek consultation with an orthopaedic surgeon, particularly those specialising in knee pathology. The aetiology of knee pain is diverse, as the knee is highly susceptible to trauma and various degenerative, inflammatory, and mechanical conditions [34]. Common causes include osteoarthritis, meniscal or ligamentous injuries, and fractures [4, 34], which comprise the majority of cases in a knee surgeon's practice.

Less commonly, knee pain may originate from hip pathology. The anatomical link between the hip and knee has been described by Sakamoto et al. [31], with sensory innervation from the femoral and obturator nerves contributing to pain referral. This phenomenon, known as ‘referred pain’, has been documented in various studies but remains incompletely understood. The most widely accepted explanation is Ruch's convergence‐projection theory, which suggests that pain may be perceived in a location distinct from the actual site of pathology due to neural convergence and misinterpretation by the central nervous system [14, 24, 29, 30, 32].

Referred knee pain is well recognised in paediatric orthopaedics, particularly in conditions such as Legg–Calvé–Perthes disease and slipped capital femoral epiphysis [2, 12, 18, 22], where it can lead to diagnostic challenges. However, in adult orthopaedic practice, this phenomenon has been less extensively studied, with only a limited number of reports and case series addressing the matter. Existing studies primarily focus on the hip surgeon's perspective, examining whether hip osteoarthritis contributes to knee pain [1, 5, 7, 15, 26, 31, 37]. One study reported that 16% of patients undergoing total knee arthroplasty for knee pain had an underlying ipsilateral hip pathology [1].

The primary aim of this study is to quantify the incidence of referred knee pain from hip pathology in the routine practice of a knee surgeon. Secondary aims include identifying common hip pathologies, treatment outcomes, and subgroup differences by sex and age. It is hypothesised that referred knee pain remains underrecognized, with an incidence exceeding 10%. To the knowledge of the authors, this is the first study to quantify the incidence of referred knee pain in the routine practice of a knee surgeon, providing novel clinical evidence from this perspective, especially in light of recent findings on non‐knee sources of knee pain such as lumbar pathology.

## MATERIALS AND METHODS

A retrospective observational study was conducted of all patients presenting with knee pain for their first consultation at the orthopaedic service. The study included self‐referred patients, referrals from general practitioners, and second‐opinion consultations. Patients were filtered based on the chief complaint of knee pain documented in referral notes or self‐reported history; ambiguous complaints (e.g., thigh pain) were included if localised to the knee during initial assessment. All consecutive first‐time consultations for knee pain (*n* = 1000) during the study period were included, representing the natural cohort size rather than a pre‐specified target. No formal sample size calculation was performed, as this was a descriptive retrospective study powered by the observed incidence; post‐hoc analysis confirmed adequate precision (95% confidence interval for 13.7% incidence: 11.5%–16.0%) [35]. All patients were assessed by a senior orthopaedic surgeon specialising in sports injuries, degenerative knee surgery, and revision knee surgery. The review period spanned from September 2023 to September 2024.

Exclusion criteria included prior ipsilateral hip surgery (e.g., total hip arthroplasty) or known spine pathology contributing to pain. All ages were included to capture the full spectrum of presentations.

During the initial consultation, a comprehensive patient history was obtained, followed by a standardised physical examination of both the knee and hip. Hip assessment included gait analysis, range of motion evaluation, and specific provocative tests, such as the flexion adduction internal rotation (FADIR) test, flexion abduction external rotation (FABER) test, log roll test, Thomas test and palpation of the groin [16, 21, 25, 28]. All examinations were performed by a senior orthopaedic surgeon specialising in sports injuries, degenerative knee surgery, and revision knee surgery. These tests demonstrate moderate to high inter‐rater reliability (kappa 0.6–0.9) in clinical settings for detecting hip pathology.

Patients suspected of having hip pathology were referred to one of two hip specialists at the same hospital for further evaluation. Treatment was individualised and included conservative management (physiotherapy and/or intra‐articular corticosteroid injection) or surgical intervention when indicated [17, 23]. Physiotherapy was targeted at the hip joint, focusing on strengthening and mobility, though some included lower limb exercises. Hip preservation procedures, such as hip arthroscopy or peri‐acetabular osteotomy (PAO), were performed in selected cases, while total hip arthroplasty (THA) was indicated for patients with end‐stage osteoarthritis.

Following treatment, patients were reassessed by the hip specialists. Due to the retrospective design, visual analogue scale (VAS) scores were not systematically recorded in consultation reports (<10% of cases). In the majority of cases, a subjective improvement in pain, as reported by patients during follow‐up, was considered a positive outcome for diagnosing referred knee pain. When available, a ≥ 50% reduction in VAS score (from baseline) or complete resolution (VAS = 0) was used to confirm the diagnosis [13].

### Statistical analysis

Data were analysed descriptively using means ± standard deviations (SD) for continuous variables and frequencies/percentages for categorical variables. Inferential statistics included chi‐square tests for categorical comparisons (e.g., sex differences) and independent *t*‐tests for continuous variables (e.g., age subgroups), with significance set at *p* < 0.05. Analyses were performed using SPSS version 28.0. Consultation with a biostatistician ensured appropriate methods for this observational design.

#### Ethical aspects

This study was approved by the Institutional Review Board of the participating hospital. Due to the retrospective design, informed consent was waived.

## RESULTS

Between 1 September 2023 and 31 August 2024, a total of 3392 patients were seen in knee consultation at the hospital (including follow‐ups). Patient demographics for the study cohort are summarised in Table 1.

**Table 1:** Demographics of first‐time knee pain consultations.

Category	Number	Percentage % – range
Total number of patients	3392	
First time	1000	28.5%
Gender		
Male	471	47.1%
Female	529	52.9%
Mean age	51.8 ± 15.2 years	25−92 years
Referral		
Own initiative	495	49.5%
General practitioner	271	27.1%
Second opinion	234	23.4%

Of these, 1000 patients (28.5%) were first‐time consultations for knee pain. Among this group, 185 patients (18.5%) were referred to the hip unit. A confirmed diagnosis of hip pathology was established in 157 patients (15.7%). The distribution of diagnoses and treatments is detailed in Table 2.

**Table 2:** Diagnosis and treatment.

Catergory	Number	Percentage % – range
First time	1000	
To hip unit	185	18.5%
Hip pathology	157	84.9%
No hip pathology	28	15.1%
◦Spinal	7	3.8%
Pathology		
FAI	36	22.9%
Dysplasia	7	4.5%
Osteoarthrosis	91	58.0%
Mixed	23	14.7%
Combined knee and hip	27	17.2%
Treatment		
Physiotherapy	47	29.9%
Corticoïd infiltration	85	54.1%
Hip arthroscopy	10	6.4%
PAO	3	1.9%
THA	12	7.6%

Abbreviations: FAI, femoroacetabular impingement; PAO, peri‐acetabular impingement; THA, total hip arthroplasty.

Among patients seeking a second opinion, 12% were diagnosed with hip pathology. Notably, among new patients referred by a general practitioner, 14.8% had an underlying hip pathology.

**Figure 1:**
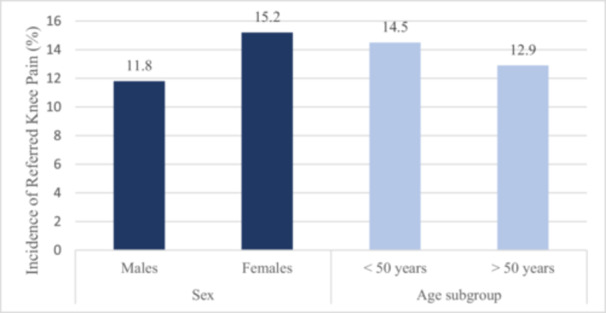
Bar chart showing incidence of referred knee pain by sex and age subgroup (*n* = 1000; *p*‐values as above).

The demographics of the referred patients are presented in Table 3. In total, 137 (13.7%) of the 1000 new patients evaluated for knee pain were diagnosed with referred pain originating from the hip or spine, rather than intrinsic knee pathology. Subgroup analysis revealed that referred knee pain was more prevalent in females (15.2% vs. 11.8% in males; χ² = 3.4, *p* = 0.03) and in patients under the age of 50 years (14.5% vs. 12.9% above 50 years; χ² = 12.1, *p* < 0.001), as shown in Figure 1. In patients <50 years (*n* = 512), FAI was most common (32.4% of hip pathologies); in patients >50 years (*n* = 488), osteoarthritis predominated (72.5%; χ² = 12.1, *p* < 0.001; age difference *t* = 4.2, *p* < 0.001).

**Table 3:** Demographics referred patients.

Catergory	Number	Percentage % – range
First time	1000	
Hip pathology	157	
Gender		
Male	55	35.0%
Female	102	65.0%
Mean age	48.1 ± 12.5 years	25−88 years
Referral		
Own initiative	89	56.7%
General practitioner	40	25.5%
Second opinion	28	17.8%

## DISCUSSION

The most important finding of the study was that referred knee pain from hip pathology was identified in 13.7% of patients presenting for first‐time knee pain consultations.

In this 12‐month study, we observed that a wide range of knee and hip pathologies prompted patients with knee pain to seek consultation with a knee surgeon. Among 1000 new patients, a significant proportion (18.5%) were referred to a hip specialist. While not all experienced complete resolution of knee pain, the majority showed improvement following appropriate management, confirming the diagnostic value of a comprehensive assessment that includes the hip. Subgroup analyses indicated sex differences, with higher prevalence in females (*p* = 0.03), and age‐related patterns, with FAI more common in younger patients and osteoarthritis in older ones (*p* < 0.001) [6, 9, 27]. Ethnicity and physical activity data were not systematically collected in this retrospective cohort, limiting further analysis.

These results support earlier reports suggesting that hip pathology can masquerade as knee pain, potentially leading to misdiagnosis and unnecessary interventions. For instance, Emms et al. and Dibra et al. described cases where hip osteoarthritis was initially misdiagnosed as primary knee pathology, resulting in ineffective treatments [5, 7]. Similarly, Gannon and Gustilo reported cases where treating the hip, rather than the knee, resolved persistent knee pain [8].

Despite this evidence, referred knee pain remains underrecognized, particularly in general and sports medicine settings. This limited recognition is problematic, as clinical knee examinations often omit systematic hip assessments, leading to diagnostic delays or incorrect management [4, 34]. Recent literature reinforces this, showing that lumbar spine pathology can also refer pain to the knee, emphasising the need for broader differential diagnosis [3]. In this study, 13.7% of new knee pain patients were ultimately diagnosed with referred pain, which closely aligns with previous findings. Lesher et al. showed that up to 22% of patients with hip pathology had referred knee pain, and Wang et al. reported that ipsilateral knee pain improved in most patients following total hip arthroplasty (THA) [20, 37]. Additionally, recent editorial commentary highlights how ipsilateral knee pain negatively affects hip arthroscopy outcomes, supporting the call for integrated assessments [33]. Ruch's convergence‐projection theory provides a physiological explanation, suggesting central misinterpretation of nociceptive input due to neural convergence in the spinal cord [29].

However, the clinical implications of this phenomenon remain insufficiently addressed. Referred knee pain constitutes a significant diagnostic blind spot that can result in unnecessary imaging, physiotherapy, injections, or even surgery. Failure to recognise it as a legitimate health problem may burden both patients and healthcare systems. These findings emphasise the need for clinicians to adopt a more integrative approach. Systematic inclusion of hip assessments (e.g., FADIR, FABER, log roll, and Thomas tests) during knee evaluations could reduce diagnostic error and prevent ineffective treatments.

To incorporate these findings into clinical practice, structured screening protocols and updated diagnostic guidelines are needed, such as recent algorithms for prescribing conservative treatment in hip‐related pain [36]. Education of general practitioners and orthopaedic trainees should stress the importance of considering extrinsic sources of joint pain. Multidisciplinary collaboration with hip specialists, as demonstrated in this centre, may further enhance diagnostic accuracy and treatment outcomes.

In cases of combined pathology, treatment was prioritised based on clinical judgement: hip intervention first if diagnostic hip injections resolved knee pain or if hip symptoms predominated; otherwise, knee treatment was addressed sequentially.

Additionally, these results reinforce the relevance of prior reports. Atanda et al. described how paediatric hip disorders often present with knee pain, and Poppert and Kulig illustrated a similar case in adults [2, 26]. Recent analyses of referred pain locations in paediatric hip disorders align with these observations, underscoring consistent patterns across ages [19]. Al‐Hadithy et al. raised concern that some patients undergo total knee arthroplasty for pain not originating from the knee, underscoring the potential for inappropriate surgery [1].

Several limitations warrant discussion. The retrospective design relies on existing clinical documentation, which may introduce reporting bias. Notably, VAS scores were inconsistently documented (<10% of cases), necessitating reliance on subjective pain improvement in most cases, which may reduce diagnostic precision. The inclusion of all ages may bias results toward different pathologies, and BMI, ethnicity, and physical activity data were unavailable, limiting subgroup analyses. The study's single‐centre setting limits generalisability, although the findings are consistent with international literature. Additionally, the systemic effects of intra‐articular corticosteroid injections in the hip remain an important confounder. Although intended for localised action, corticosteroids exhibit systemic absorption that may influence pain perception in distant joints, including the knee [10, 11, 23]. Further prospective studies are necessary to clarify the exact mechanisms of referred pain and to determine whether interventions such as hip injections may inadvertently mask knee symptoms, or vice versa.

## CONCLUSION

Referred knee pain from hip pathology is a prevalent and underrecognized phenomenon, affecting 13.7% of patients in a knee surgeon's practice. Routine hip evaluation in knee pain assessments is essential to prevent misdiagnosis and unnecessary interventions, as multidisciplinary collaboration has been shown to improve diagnostic and treatment outcomes in this single‐centre study; multi‐centre validation is warranted.

## AUTHOR CONTRIBUTIONS

Bert Cornelis is the main author. Gilles Van Eetvelde, Sigurd Uyttebroek, Maxence Vandekerckhove, and Jan Vanlommel contributed to the data gathering and review of the article. Pieter‐Jan Vandekerckhove served as the supervisor of the project.

## CONFLICT OF INTEREST STATEMENT

The authors declare no conflicts of interest.

## ETHICS STATEMENT

Approval was given by the ethical board of AZ Sint Jan Brugge (IRB approval number: 2023‐045).

## Data Availability

Data available on request due to privacy/ethical restrictions.
